# Perspectives on preventing musculoskeletal injuries in nurses: A qualitative study

**DOI:** 10.1002/nop2.272

**Published:** 2019-04-13

**Authors:** Amy Richardson, Gagan Gurung, Sarah Derrett, Helen Harcombe

**Affiliations:** ^1^ Injury Prevention Research Unit, Department of Preventive and Social Medicine, Dunedin School of Medicine University of Otago Dunedin New Zealand; ^2^ Department of Preventive and Social Medicine, Dunedin School of Medicine University of Otago Dunedin New Zealand

**Keywords:** intervention, musculoskeletal, nurse, nursing, pain

## Abstract

**Aims and objectives:**

To explore the perspectives of nursing and physiotherapy academics regarding techniques designed to prevent musculoskeletal pain and injury in nurses.

**Background:**

High rates of musculoskeletal injuries are evident in nurses, yet there is an absence of research identifying effective interventions to address this problem. Exploring the perspectives of individuals with specialist knowledge in the area could help identify barriers to musculoskeletal injury prevention, and innovative strategies to investigate in future studies.

**Design:**

Cross‐sectional qualitative descriptive study.

**Methods:**

Between October–December 2017, group and individual face‐to‐face semi‐structured interviews were used to collect data. All interviews were audio‐recorded. A thematic analysis was performed, with two researchers coding audio files using NVivo software. The Consolidated Criteria for Reporting Qualitative Research Checklist was consulted to ensure complete reporting of all methods and findings.

**Results:**

Nursing and physiotherapy academics (*N* = 10) were aware of a range of techniques to prevent musculoskeletal injuries in nurses, including education, equipment, health and safety policy and multi‐disciplinary collaboration. However, several barriers to using these techniques were identified, including age, knowledge and availability of equipment, personal and contextual factors, staffing and time pressures. Several strategies were recommended for further investigation and implementation in clinical practice, such as the sharing of personal experiences, orthopaedic assessments and changes to workplaces that foster a culture of safety.

**Conclusions:**

Further research is required to reduce musculoskeletal pain and injury among nurses. This research should account for the barriers to current prevention strategies and consider investigating novel interventions.

**Relevance to clinical practice:**

These findings highlight strategies for preventing musculoskeletal injuries among nurses that are likely to be most effective in clinical practice.

## 1 INTRODUCTION

High rates of back injuries and pain have long been identified in nurses (Harcombe, McBride, Derrett, & Gray, 2009; Jensen, 1987; Trinkoff, Lipscomb, Geiger‐Brown, & Brady, 2002). A review of over 80 studies found low back problems in nurses had a point prevalence of approximately 17%, annual prevalence of 40%–50% and lifetime prevalence of 35%–80% (Hignett, 1996). According to the United States (U.S.) Bureau of Labor Statistics, rates of injury and illness for nurses rank among the highest across occupations, including most manufacturing, construction and agricultural sectors (Bureau of Labor Statistics, 2016). In New Zealand (NZ), the lifetime prevalence of back pain among nurses is estimated to be 74% (Coggan, Norton, Roberts, & Hope, 1994).

Risk of experiencing pain and injury increases with the number of years that nurses spend in the profession. A prospective cohort study of 175 nursing students in Australia documented a 41% increase in the lifetime cumulative prevalence of back pain, from beginning to completing nursing school (Videman, Ojajärvi, Riihimäki, & Troup, 2005). This prevalence increased further following transition into the paid nursing workforce, with 82% reporting pain after 5 years (compared with 31% reporting pain on entry to nursing school).

High rates of pain and injury experienced by nurses contribute to absences from work, inadequate staffing levels and high turnover. Survey data from 1,653 registered nurses in the United States identified that having more strains or sprains, including back injury, were a significant predictor of whether participants left their employer 1 year later (Brewer, Kovner, Greene, Tukov‐Shuser, & Djukic, 2012). Costs are associated with nursing turnover. Estimates using predominately U.S. data suggest that the cost of replacing a single nurse can range from $10,098–$88,000 (Li & Jones, 2013). In NZ, the cost of replacing a registered nurse is approximately half an average nurse's salary ($23,800), with the greatest expense attributable to temporary cover costs, followed by orientation/training and lost productivity (North et al., 2013).

Manual handling of patients has been identified as a key contributor to musculoskeletal injuries and pain among nurses (Pompeii, Lipscomb, Schoenfisch, & Dement, 2009). Nurses are required to move and reposition patients on a daily basis which can involve awkward postures (stooping, bending and reaching), use of manual force and repetitive actions (Choi & Brings, 2016). Nurses who experience moderate or high physical demands are at greater susceptibility of neck, shoulder and back injuries than those with low physical demands (Trinkoff et al., 2002), even when adjusting for demographic and lifestyle factors (Trinkoff, Brady, & Nielsen, 2003).

The occurrence of musculoskeletal injuries and pain in nurses has also been related to other factors, including inadequate staffing, unpredictable work hours and fatigue (Vendittelli, Penprase, & Pittiglio, 2016). For example, perceiving staffing as inadequate was associated with a 64% increased risk of back pain in a cross‐sectional study of 1,345 intensive care nurses in South Korea (June & Cho, 2011). Cross‐sectional and longitudinal research with nursing students in Australia highlights the role of personal factors such as age, smoking, physical activity, stress levels, coping, muscle endurance and spinal positioning in the development of low back pain (Mitchell et al., 2009,2010).

Efforts to reduce the high prevalence of pain and musculoskeletal injuries among nurses include “no lift” policies, injury prevention guidelines, the provision of equipment and manual handling education (Hunter, Branson, & Davenport, 2010). However, there is substantial variation in the application of injury prevention efforts across different hospitals. In a recent survey of 72 registered nurses working in hospital inpatient units in the United States, only 14% of participants stated a written “no lift” policy had been implemented in their workplace (Vendittelli et al., 2016). Adequate staffing for the performance of safe patient manual handling was reported by just 33% of participants, and only 39% believed their workplace had all of the equipment needed to perform manual handling tasks safely.

In addition to inconsistencies in implementing strategies to prevent musculoskeletal injuries in nurses, there exists limited empirical evidence to support the effectiveness of such strategies (Hignett, 2003). The most recent relevant systematic review identified that although many interventions have been tested, no strong evidence for any intervention has been found (Richardson, McNoe, Derrett, & Harcombe, 2018). These findings suggest that it may be important to investigate alternative interventions, using designs that can account for the many factors known to play a role in the development of musculoskeletal injuries among nurses.

There is an absence of qualitative research on the perspectives of nurses and other professionals regarding techniques currently used to prevent musculoskeletal injuries and additional approaches worthy of investigation (Hignett, 1996). However, examining the views of individuals with expert knowledge of methods to prevent musculoskeletal injuries in the training and working environments of nurses may help to identify interventions that are likely to be most effective and practical to implement in clinical practice.

## 2 THE STUDY

### 2.1 Aims

The aim of this study was to investigate nursing and physiotherapy teaching academics' perspectives on the effectiveness and use of techniques designed to prevent musculoskeletal injuries among nurses in NZ, including approaches they believed to be worthy of future research or implementation in clinical practice.

### 2.2 Design

This study used a cross‐sectional qualitative descriptive design (Sandelowski, 2000) and was guided by a realist epistemological framework. Qualitative description is recommended to generate straightforward descriptions of the topic under investigation and involves interpreting data based on the semantic (or surface) content of words and phrases used by participants (Sandelowski, 2000).

### 2.3 Sample

A mixture of purposive and snowball sampling was used to recruit participants. Heads of Department from a School of Physiotherapy and a School of Nursing were contacted and asked to help identify suitable potential participants. An information sheet describing the study was sent to these staff members with a consent form. Interested individuals indicated their willingness to participate via email. Participants were asked for suggestions of additional potential participants.

Eligible participants were teaching and/or conducting research in the fields of physiotherapy or nursing. Additional inclusion criteria for physiotherapy staff included having a teaching or research focus on musculoskeletal injury prevention and/or familiarity with the clinical context of nurses working in NZ; and for nursing staff having prior experience working as a registered nurse in NZ.

A sample of 10 participants was identified as sufficient for data saturation, when it was deemed by the researchers responsible for data collection that no new information was being attained. Participants had limited time and therefore, for pragmatic reasons, participants were offered a choice of taking part in a group or an individual interview.

### 2.4 Data collection

Three group interviews and two individual interviews were conducted between October–December 2017. All interviews occurred in one region of the South Island of NZ, to ensure that participants could easily attend an in‐person interview. Interviews were conducted by one female and one male interviewer (AR and GG) in a private room. One interviewer was responsible for asking the interview questions (AR) and one made field notes (GG). Both interviewers had prior experience undertaking qualitative research.

Interviews were semi‐structured and ranged from 31–71 min in duration. Participants first provided a brief description of their background in nursing/physiotherapy and their current role. Participants then answered open‐ended questions regarding their perspectives on techniques to prevent musculoskeletal injuries and pain among nurses (Table 1). All interviews were audio‐recorded.

**Table 1:** Interview guide

Order	Question
1	We know the incidence of musculoskeletal disorders is high among nurses. What do you think could be done to reduce this?
2	What techniques are you are aware of that are used to prevent musculoskeletal injuries and pain among nurses?
3	What techniques to prevent musculoskeletal injuries and pain are most effective?
4	What techniques to prevent musculoskeletal injuries among nurses are currently encouraged in New Zealand hospitals?
5	To what degree are current prevention techniques utilised by nursing staff? Why/why not?
6	Are there any other techniques/methods/approaches for protecting nurses from musculoskeletal injuries that you think should be adopted? Why/why not?
7	Results of international studies suggest that there is limited empirical evidence for interventions aiming to reduce musculoskeletal injuries in nurses. Which approaches do you think are most worthy of further research?
8	Is there anything else you would like to say about the experience of musculoskeletal injuries among nurses?

### 2.5 Ethical considerations

All participants completed a written consent form at the beginning of each interview, and participants were informed there was no obligation to answer the questions and that they were free to leave at any time. Participants were asked not to repeat information to others after each interview had finished. The study received University ethics approval.

### 2.6 Data analysis

Data were analysed using NVivo Version 11 (NVivo, 2015). A thematic analysis was performed according to the six steps specified by Braun and Clarke (2006): familiarization with the data, coding, searching for themes, reviewing themes, defining and naming themes and write up. Themes were identified in each of the four broad areas examined across the interview: (a) strategies to prevent musculoskeletal injuries in nurses in NZ; (b) use of strategies; (c) most effective musculoskeletal injury prevention strategies; and (d) approaches worthy of further investigation.

Two researchers (AR & GG) listened to each audio recording in full to become familiar with the data, noted important features relevant to the study aims and generated succinct labels (codes) for these features. Examples of relevant data extracts related to each code were recorded in a word document and disseminated to the broader research team. Codes were then reviewed in a meeting involving all authors, and consensus was reached regarding which codes should form the basis of a working analytical framework.

The analytical framework was used to code the audio files. Two researchers (AR & GG) independently coded the first audio file to investigate whether the coding framework was being applied consistently. Average percentage agreement was calculated using an NVivo coding comparison query and areas of discrepancy (where agreement for individual nodes was <90%) were identified. Differences in interpretation were discussed in meetings and resolved. The first audio file was then independently coded by the researchers a second time, which resulted in >90% agreement for all nodes (range = 92%–98%). The remaining interviews were then coded; each interview was reviewed by the other researcher to check for completeness and accuracy. Following this, AR and GG met to collate codes and relevant data extracts into potential themes. These were checked against the dataset and then refined, with several codes combined into broader categories and others discarded. The final set of themes was reviewed by the entire research team. A detailed description of each theme was completed, with supporting quotes provided.

### 2.7 Rigour

The use of NVivo software has been found to enhance qualitative research by allowing for greater efficiency and transparency when analysing and reporting data (Hoover & Koerber, 2011). Detailed notes on impressions of each individual interview and the coding process were made by the researchers when completing the analysis; these are available on request. The use of two researchers to analyse the data increased the rigour of this study, by ensuring that multiple interpretations of the data were considered throughout the coding process (Barbour, 2001). The Consolidated Criteria for Reporting Qualitative Research Checklist and Standards for Reporting Qualitative Research were consulted to ensure the complete reporting of all relevant methods and findings.

## 3 FINDINGS

### 3.1 Participants

The study had 10 female participants; five physiotherapy and five nursing academics. One nursing and one physiotherapy participant were individually interviewed. The remaining interviews were group interviews: two involved two physiotherapy academics and one involved four nursing academics. Quotes from physiotherapy participants are denoted with a “P” and nursing participants with an “N.”

### 3.2 Strategies to prevent musculoskeletal injuries in nurses

Participants were aware of several strategies to prevent musculoskeletal injuries in nurses, which were largely described in relation to the NZ context. Key themes that emerged included education, equipment, health and safety policy and multi‐disciplinary collaboration.

#### 3.2.1 Education

Education was one of the most frequently identified strategies and included descriptions of the manual handling training provided to nursing students, manual handling training for nurses in clinical contexts and education regarding risk assessment.

Participants were aware of manual handling training for nursing students that is provided towards the end of the first year of tertiary education prior to hospital placements beginning the following year. This training involves a 2‐hr long practical session conducted by physiotherapists. Participants explained that the training provides students with basic knowledge of how to prevent musculoskeletal injuries when performing patient transfers. However, participants noted there was a lot to cover in a single session and that some students might not be aware of the importance of the training for their future roles as nurses:

Sometimes they don't get the fact that actually this is a skill that you need for your clinical practice… they don't quite relate [it] to what they're going to be doing in their clinical practice. (P2)

In addition to the manual handling training provided to nursing students, it was noted that healthcare facilities provide manual handling training as part of the orientation process for new nursing staff. However, the application of such training was considered highly variable. Participants highlighted that many organizations now provide manual handling training for their employees online: “It was only online, in fact, there was no practical component. I was actually never shown how to use a hoist…” (N2).

Several participants identified risk assessment as an important aspect of the manual handling training provided to nurses. This includes encouragement to consider hazards in the environment that are likely to pose an injury risk. While risk assessment was described as an important component of the education provided to nurses, participants acknowledged that it was not necessarily sufficient to prevent musculoskeletal injuries:

We look at risk, risk assessment and I do stress a lot of that during the teaching of the overall workplace they're in and then of the specific tasks that they've got to carry out… it's that total package… but still we know that, you know, injuries occur. (P3)

#### 3.2.2 Equipment

Participants described the provision of equipment as a strategy to prevent musculoskeletal injuries among nurses. Equipment is designed to assist in the transfer and lifting of patients (e.g., sliding sheets, rota stands, hoists). Some participants felt that equipment had become more readily available in NZ when compared to previous years:

The general hospital is becoming more aware of preventing injury and there are, there is more equipment available in some areas, like those rota stands… I have seen over the last few years that things like that are becoming much more common than they were. (P5)

However, participants were unaware of any processes in place to assess competency among individuals using equipment. One academic commented: “I don't know if I've ever had any induction into lifting or handling coming into the environment… as a visiting clinical supervisor” (P4).

#### 3.2.3 Health and safety policy

Several participants identified health and safety policy as a strategy used to prevent musculoskeletal injuries in nurses, including the introduction of Accident Compensation Corporation (ACC) guidelines. ACC is NZ's universal no‐fault accidental injury scheme, providing cover for rehabilitation and support associated with injury. Some participants believed these guidelines had made an important contribution to injury prevention by ensuring that employers were held accountable for the safety of their staff:

ACC guidelines really did bring the employer on board… it really has helped. And it's helped the employee understand that they have a responsibility, too, to their own safe practice and updating their knowledge. (P3)

Participants noted that ACC and other health and safety guidelines encourage a “no lift policy”:

That's health and safety, across the board, no lift – but we slide, we transfer and we support and guide… and get the patient to do as much as they can. (P3)

While some participants considered no lift policies to have led to greater availability and use of lifting equipment, others were not convinced:

I mean, I know the latest code is about no lifting – it's about moving and handling, not lifting and handling, because you're not allowed to lift a patient – you use a hoist nowadays… which is a move away from “yeah we just do it”. The problem is nurses tend to do what they do without thinking. (P1)

Furthermore, some participants believed that no lift policies do not provide a clear indication of approaches that should be taken instead:

Focusing on, like, it is a no lift, not to lift patients, but I don't think it really goes into specifics… like this is what to do if your patient is really heavy and you need to move them. (P5)

#### 3.2.4 Multi‐disciplinary collaboration

Multi‐disciplinary collaboration was identified by some participants as a strategy to prevent musculoskeletal injuries:

I generally do think that the nurses are encouraged if they're unsure about somebody's mobility to contact a physio to assess them to say this would be the best way to transfer. (P5)

However, this collaboration and its associated benefits were largely realized during daytime working hours, with little opportunity for this strategy to be used outside of these hours:

Sometimes the support in the daytime hours, like physiotherapists around they're doing rehabilitation, there's a whole range of inter‐professional groups focusing on the person, but nurses tend to be everything to everybody after 5 o'clock until 8 o'clock the next day and that's sometimes when there are, I'm sure, there is a higher potential for injury then. (N3)

### 3.3 Use of strategies to prevent musculoskeletal injuries

#### 3.3.1 Use of equipment and manual handling techniques

The visibility and accessibility of certain types of equipment was thought to contribute to use in hospitals, particularly sliding sheets and boards:

You can keep the “pat board” in your room, in a four bedded room, keeping it safe down in the corner somewhere and you can have your “slippery sam” folded and ready to use in the patient locker beside the bed. (N4)

This type of equipment was also perceived to save time, resulting in greater use: “definitely easily accessible, time wise they are an easier solution” (N2). However, not all participants agreed on the degree to which equipment was available:

Things like the “slippery sams” are not well utilised… it tends to be there's “slippery sams” available but they're kept in a box so you're going to need to go find one. (P5)

There was consensus that the ease of using equipment contributed to whether or not it was actually used: “Some of these smaller pieces of equipment, the “slippery sams” are amazing and these… rota frames… its easy, it's easier to use them than not use them” (P2).

Variation in the use of equipment was believed to occur across different clinical contexts. Participants felt that hoists were regularly used in aged care settings. This was described as partly attributable to inadequate staffing:

and because the carers and nursing staff there in the rest home environment… they're really understaffed and so they often have to transfer on their own and so their go to is to use a standing hoist (P4)

—but also patients' greater mobility needs.

Participants had differing opinions regarding the application of manual handling techniques, although those who believed that they were well used were in the minority:

I certainly see them being utilised. Equally I have seen older nurses still employing some pretty old techniques because its quicker… but the majority of times, I would say 95% of the times, I see really good technique[s]. (N1)

Most participants felt unsure about whether or not manual handling skills acquired during training sessions were subsequently applied in clinical practice:

You'd be lucky if 50% actually do it… If they followed through with what we said, yeah, I think it would be a benefit, but that's outside my control. (P1)

#### 3.3.2 Barriers to use

A diverse range of barriers to the use of musculoskeletal injury prevention strategies was identified by the participants, including availability of equipment, age, personal factors, contextual factors, staffing and time (Table 2).

**Table 2:** Barriers to utilization of strategies to prevent musculoskeletal injuries among nurses

Theme	Sub‐themes	Example quotes
Availability of equipment	Limited availability	“Certainly a couple of years ago they didn't even have the patient sliders… they didn't even have them and so it's quite difficult” (P2). “There's lots of room for improvement, masses of room for improvement” (P3)
	Absence of electrical beds	“Some of the wards don't even have electric beds so you know the nurses are kind of at a starting point to get injured anyway cause if you've got a dead weight of a patient whose got no active moment, to actually move the bed from flat to 30 degrees uses all your man power” (P5). “Before that I was working [overseas]– they had all electric beds and actually their old beds they were, like, sending to third world countries so when I came back… it felt like a third world country” (P5)
	Absence of specialist equipment	“I was looking after this hugely obese man… he was too big for the hoists. We literally had to get on the bed and push him. We had to do it manually… there was no piece of equipment available” (N5). “Finding belts that are big enough is an issue these days… sometimes it's like there's no way that's going to fit round this person” (P4). “I think we are ill‐equipped and we're seeing more and more of those kind of patient populations” (N2)
	Difficulties locating equipment	“Finding a walking frame is sometimes impossible” (P4). “If it's not readily available, it's too hard to go and locate cause it's not somewhere accessible… it's easier to do a dodgy transfer than go and find where the piece of equipment is cause that's seen as wasting time” (P5)
Age	Relationship with physical fitness	“Dare I say it with some of the older nursing fraternity; they're not particularly physically fit” (P1)
	Prior experience	“Some of the nurses that work in the hospital probably graduated and worked before no lift policies came into play and so they're very much “I've always done it like this, it will be fine”… as opposed to the newer cohort of nurses who have been following what the guidelines are… I think they're a lot more protective of looking after their backs” (P5)
	Ingrained attitudes	“The older nurse… has been doing whatever they've been doing a certain way for a very very long time. I mean, I've been doing manual handling training and actually certain nurses have refused to do it – “oh it's a waste of time, I don't wanna do it”” (P1)
Personal factors	Beliefs/attitudes	“There is a group of people who think… “oh, I'll do it my way”” (P5). “You know, the not lifting over 15kgs… it's been in for a long long time and people still do it. Next thing they say “my back”” (N1)
	Altruism	“I'm generalising but I don't think we always think of ourselves when we're doing something – it's about the best outcome for the patient and so I think that sometimes we put ourselves in vulnerable positions that we don't intentionally do, and sometimes injuries arise from those positions” (N3)
	Weight and physical fitness	“You can see some who are really fit and you can see some who are quite overweight… you do wonder how they're going to manage to perform correct techniques that they have to during their practice” (P3)
Contextual factors	Demands from other staff members	“You've got all the good intentions in the world but you've got time pressures, you've got someone yelling at you here, you've got a consultant saying you need to come and do this, you've got the head nurse saying someone's bled out over here…” (P1)
	Hierarchies	“Consultants have just gone at them and they're left feeling alone, victimised, bullied, all of those sorts of things so that creates stresses in your work place which leads on to other health issues” (P1)
	Workload	“Generally workloads are getting higher” (P3). “The real pressure occurs in the evening. I mean during the day there's a lot of people around, people tend to be fresher, not in so much pain, they don't get so tired but as you get through the evening they get really quite distressed and sore” (N4)
	Modelling	“Once you see bad habits and they've been demonstrated without any ill effect, they're easy to pick up. It's human nature…” (N1)
	Ward culture	“Some of the charge nurses are very into preventing injury and so if there was maybe special equipment that the ward didn't own that you could actually hire to use just for this patient some wards would be very happy to say well if that's the best piece of equipment for this person and it's gonna be safe for everybody they'll pay the cost and hire it and other wards will be like “no we're not hiring anything” so you've got to make do with what you have… that's obviously a resource issue as well but it's almost a culture thing” (P5)
	Emergency situations	“All the good advice goes out the door” (P5). “If you were in a life or death situation people may panic and not really consider themselves… it's that fight or flight thing: you can lift people, you can move bridges, you can do anything you like in that situation, but I do believe you could put yourself at risk completely unwittingly” (N1)
Staffing	Insufficient staff numbers	“That more likely to do it yourself approach because you've got less manpower available” (N2). “There's low staff and therefore… the staff are so pressured they just go the quickest way which might be using less staff than is recommended because it's too hard to get that many people to come and help. I would say it's one of the biggest issues and there's not that much resource at hand and then there's also urgency to get people up and moving” (P5)
	Staff unavailable to provide training	“That often comes back to staffing because having the time to release the staff to do some training or having time to go around the staff and make sure they understand the equipment, well it's not there” (N5)
Time	Time pressure	“On the wards and stuff time pressure is massive, you don't need anything that's gonna add an extra 20 min to your schedule” (P2). “I don't think nurses have bad intentions, I just think they've got so much other stuff going on that if this could be a shortcut and its gonna save 10 min and you're already having a busy day… “okay I'll just do it like this”” (P5)
	Time‐consuming equipment	“It's quicker to get two people to hoist the person up the bed than it is to get the machine or hoist in to strap them all up and move them up it” (N5). “If you need to go and find some equipment, even if it's just a ‘slippery sam' or something like that, as well if you're busy it's like well let's not do that” (P4)
	Pressure from other staff members	“Trying to complete your nursing tasks in a given time could lead to errors in lifting and transferring… I hurt myself because this person wouldn't give me the time to make the adjustment and as a result I had to have some surgery… and it really brassed me off because a two minute adjustment was all I needed to do” (N1)

##### Availability of equipment

Not all equipment that could help to prevent musculoskeletal injuries was perceived to be available by the participants. Participants were particularly concerned about the absence of electrical beds in hospitals and the injury risk this might be posing for nurses. Participants also reported a notable absence of specialist equipment, particularly equipment that could assist with lifting and moving obese patients. Participants expressed that difficulties with locating equipment served as an additional barrier to its use. Sharing equipment across multiple wards resulted in reduced equipment available, at times leading nurses to place themselves at risk of musculoskeletal injury.

##### Age

Most participants perceived older nurses to be less likely to engage with strategies to prevent musculoskeletal injuries than younger nurses. This was partially attributed to their being less physically fit, but also to their prior experience, including less exposure to manual handling training and equipment.

##### Personal factors

Participants felt there are some nurses who believe that strategies designed to prevent injuries are not worth using. One participant felt that some nurses perceived themselves to be immune to injury. Other participants felt that nurses often act selflessly, putting the needs of patients before their own and increasing their injury risk. Weight and physical fitness were additional personal factors thought to influence whether nurses used strategies to prevent injuries.

##### Contextual factors

Participants identified numerous factors that nurses must contend with in their work environment that make it difficult for them to use manual handling strategies and equipment, including demands from other staff members. Participants also noted the influence of hierarchies on nurses. Participants described the heavy workload of nurses, which was thought to be even greater for those working in the evenings. Techniques modelled to nurses in their work environment were thought to influence their behaviour. Similarly, the overall culture of a ward was described as having potential to be a facilitator or barrier to the use of safe techniques. Charge nurses were considered to have some control over the culture of their wards. However, emergencies were identified as a contextual factor that make it very difficult for nurses to engage with injury prevention strategies.

##### Staffing

Inadequate staffing levels were acknowledged as a barrier to engagement with effective strategies, requiring nurses to use approaches that could be performed alone. Inadequate staffing also necessitated nurses working more quickly. Inadequate staffing was perceived to result in nurses not receiving appropriate training in the use of equipment.

##### Time

Participants were all in agreement that time pressures prevented nurses from using injury prevention strategies. Saving time was believed to be a top priority of nurses. Lifting equipment was often perceived as too time consuming to use. Participants were of the opinion that even smaller aids would not be used if this were to add time to an already busy schedule. Participants felt that pressure from other staff to perform tasks efficiently was also an issue.

### 3.4 Most effective musculoskeletal injury prevention strategies

Participants considered the most effective strategies available to prevent musculoskeletal injuries among nurses to be adequate staffing, education, enforcement of policies and procedures and the promotion of physical fitness and overall health.

#### 3.4.1 Adequate staffing

Ensuring adequate staffing was considered one of the most effective ways to prevent injuries by making it possible for nurses to “divide the workload” (N2). This was thought to reduce the burden placed on individual nurses—“having multiple people if there is someone needing to be transferred so that they're not lifting as much load” (P4)—and target one of the key contributors to injury: “Staffing levels… cause often people will get injured because they're short of staff and so they're rushing or there's just not enough of them around to manage a situation” (P2).

#### 3.4.2 Education

Many participants felt that education was the most effective way to help prevent injuries among nurses:

Insisting correct techniques are used right from day one of education. Raising the beds to the correct height, not over‐extending, being positioned correctly, using any apparatus available whether it's a belt or hoist or any aid. (N1)

Experiencing lifting and handling from the patient's perspective was thought to be a particularly effective educational tool: “Experiencing safe lifting techniques as a practice patient gives you the confidence to know that you are doing it correctly” (N1). A review of skills on a regular basis was also believed to be important. One reason suggested for this was the limited time that student nurses in NZ spend completing manual handling training prior to working in clinical practice:

I think a lot of its reinforcement isn't it; [be]cause like in one two hour session that's a lot and they don't remember half of what it is that they've seen. So it's like being able to go out and practice in clinical and have those good practices reinforced and discussed. (P2)

Repetition of information, or reinforcement, was considered the best way to ensure that injury prevention techniques are used correctly.

#### 3.4.3 No lift policy

Several participants thought that no lift policies had led to reductions in the frequency and severity of injuries experienced by nurses in recent years, largely because of their association with the provision of effective education and equipment:

If I look back… I can remember lots of them having carpel tunnel troubles, neck and back problems and that's a lot lot less now because you've got protocols in place, recommendations to follow, more training and there's more mechanical aids used. (P3)

Furthermore, some participants felt that no lift policies were the most effective method for preventing injuries because they provide nurses with clear guidelines regarding how patients should be mobilized:

Based on what I know about nurses and human beings, probably the policies and procedures and the enforcements of them… because I think people will go away and do what they want to do whether they've been taught to or not… yeah I think the most useful has got to be policies saying this is what you must do. (P1)

#### 3.4.4 Promotion of healthy lifestyle

Several participants felt that encouraging physical fitness was likely to be a more effective strategy than other more traditional injury prevention techniques: “Overall I think it would be physical fitness… being fitter and stronger is more likely to prevent an injury” (P1). This was largely considered the responsibility of individual nurses: “Building personal capability… it's about people taking responsibility themselves for looking after themselves” (P2).

### 3.5 Approaches worthy of further investigation

Several additional approaches that might help to prevent musculoskeletal injuries among nurses were identified, including a culture of safety, manual handling training in clinical contexts, changes in workflow, ergonomic footwear, the provision of electric beds, needs analysis and the sharing of stories (Table 3).

**Table 3:** Approaches to preventing musculoskeletal injuries among nurses worthy of further investigation

Theme	Sub‐themes	Example quotes
Culture of safety	Changes in management approaches	“There's always a lot of management systems and management telling the workers what to do rather than going to the workers and saying what do we need to do that's going to help you” (P1). “If the staff are given the control of what needs to be put in place to make them safe they're more likely to follow through instead of management saying “this is what you must do”…” (P1). “One of the main premises of occupational health and safety is consultation and communication. Consulting with staff and asking them what they want… can't get everything, we realise that, but you make concessions” (P1)
	Routine assessments of competence	“That mantra of ensuring that people are safe and competent to use the equipment and not just kind of winging it” (N2). “There could be a practical component to your annual performance review and that is about demonstrating competence in a range of different things specific to the environment that you're working in” (N3)
	Early reporting of injury	“Get on to injuries quickly or pain quickly so that you're managing it really well – they've got access to experts in the field that can help… that could be a responsibility of the employer” (P2)
	Promotion of physical activity and healthy lifestyle	“If you make, like, tai chi classes available at lunch time or Pilates or whatever the flavour of the month is and if you make it available to your staff at knocking off time, for example, so they can do a Pilates class before they go home – you know cheaper access to gyms or whatever” (P2). “An orientation to self‐care, looking after yourself, preventing injuries, making sure that you don't put yourself in a vulnerable position… trying to just mitigate the possibilities of dangers for oneself” (N3)
	Posters and print material	“I think that there's very little information around about how to prevent injury, as in, on the other end, just to give an example, preventing falls in hospital… you can't walk in the hospital and miss it. There's posters everywhere and those posters are aimed at staff and they're also aimed at the public. I don't think I've ever seen something that says “reduce risk of injury to staff by doing A, B, C” or “have you thought about doing this?” (P5)
	Pre‐employment screening	“Pre‐employment screening may be an option – there are certain pre‐employment screening tests that have been shown to be valid to identify an increased risk” (P1)
	Hazards analysis	“I injured my back, my lower back, nursing when an oxygen cylinder, a large one, fell and it was about to hit a patient and I dived and grabbed it so that was a hazard that should have been identified and made secure so it's about a hazard analysis – looking around to prevent those kinds of things” (N4)
Manual handling training	Training within clinical contexts	“On the floor, you know, I think maybe on the floor where a physio could go round and perhaps just with the more difficult perhaps scenarios have practice sessions” (P3). “On the job training, experience, is massively important… I think perhaps in the past it was like “oh well we've done the manual handling because they did that as part of their orientation, box ticked, that's it”, but I think it's more about the integration into actual workplace practices and on the job training… I would presume that would be more effective” (P2)
	Practical experience	“It's like learning to give an injection online… you can see how to do it but until you've actually got the needle… it's the same with lifting, you can see everything but until you've actually tried it out… it has to be practical” (N4)
	Refresher courses	“Those training programmes for recertification, refresher type of thing, I think that's really important” (N1). “Refresher courses maybe every year you've gotta go to your manual handling training. It needs to be practical. I don't think watching a video is of any benefit” (P1)
	Manual handling champions	“Something that may help well is if they had, cause it's hard to expect everyone to be an expert on manual handling, but if perhaps a ward having like someone who's like a champion or spokesperson for that and if they've had extra training perhaps they could be the ones who influence the culture and thinking of the ward and bring concerns around people not utilising correct technique” (P4). “You don't tend to go and read a textbook about how to move a person from A to B, it's more about what you see people doing” (P5)
	Lift teams	“If you've got lifters available, you know orderlies or whoever available to do the lifting and they get the training, they get the proper support… and whatever they need, they're well equipped to be able to do it, do it properly and safely, that might be a potentially better way to do it” (N5)
Workflow	Well‐designed workspaces	“The physical work environment of the hospital has changed dramatically over the 30 years that I've been involved… they've got rid of clear workspaces, they've got rid of the nurses station, there's all little cubby holes and rooms… there's no process for doing things and finding things” (N4)
Footwear	Podiatrist assessment	“When you see some of the staff in hospital, they're wearing crocs, which they're actually not allowed to wear, or they're wearing canvas shoes which aren't wipe able really… or something that's fashionable as opposed to practical. And if you're working an 8 or a 12 hr shift it's no wonder that some people end up with pain and back problems when they're not actually wearing appropriate footwear. It's almost like every nurse should have a podiatrist assessment and get properly assessed in terms of what footwear they should have” (N5)
Electric beds		“That would be a really easy recommendation to stop nurses injuries is to give them electric beds. For example, especially on orthopaedics because people have lower limb injuries, they often want to elevate, you know, like their legs for swelling purposes, and actually to take that tilt off the bed is the most dodgiest thing for your back, you have to come grab the bed at the end, bend your knees and take the wait of the patient as you lower it. So that would be a really simple fix” (P5)
Needs analysis	Individual tailoring	“Tailoring whatever you're doing to the particular demands of the job… workplace assessments and setting up a work station to suit” (P2)
	Frequency of lifting across different environments	“Taking an individualised approach… people are going to act differently so not one piece of equipment is going to be appropriate. Then choosing an environment where the frequency of needing to transfer is high would probably be a better place to do it… It's not just one size fits all” (P4)
	Distribution of resources	“It is difficult for an organisation because sometimes that equipment might lie unused for days and then sometimes five people might need it at once so it is very difficult for an organisation to be able to cost wise manage that… it's just frustrating at the time when you need a piece of equipment and it's not available… so whether or not, you know, a proper needs analysis has occurred… doing a stocktake on what current equipment is available and marrying that up with the profiles of the patients that are coming into the hospitals” (N5)
Stories		“The use of stories – you give the explanations, do the demonstrations, and tell the stories of the consequences of what may happen” (N1). “Stories really are powerful” (N4). “We talk about the power of stories and perhaps actually inviting people who have encountered significant musculoskeletal injuries during their profession to inspire people to think about it a little bit more because I do think we probably brush over it” (N2). “I'm really interested in the people who have got injuries, what happened to them, what were there things, where did they find themselves, was it getting someone out of a bath, was it the day to day stuff, was it about backs wearing out. That would be interesting to then put together what kinds of things, what kinds of situations, we need to think about” (N3)

#### 3.5.1 Culture of safety

Development of a total safety culture was recognized as having great potential to prevent nursing injuries. Changes in management approaches were suggested as ways to facilitate a culture of safety. One participant felt that giving nurses greater control over the techniques they use to keep themselves safe could be beneficial. This included involving nurses in decision‐making processes. Another way to develop a culture of safety was thought to be routine assessments of competence. Participants believed that, currently, appropriate techniques are not always used correctly, which likely reduces their efficacy at preventing injury. Assessing competence in manual handling may be a way to ensure that nurses are familiar with equipment and that they know when and how to use it.

Efforts to protect the health and well‐being of nurses were thought to contribute to a culture of safety and were thought to be worthy of further investigation. This included the early detection of pain and injury so that it can be addressed before becoming a bigger problem. Encouragement to report injuries was one method that could facilitate early detection. Furthermore, the promotion of physical activity and healthy lifestyle on behalf of employers was highlighted as an additional strategy that could make a difference to the overall safety of a workplace. Self‐care could also be promoted by healthcare organizations and the effect on rates of injury among nurses investigated.

Posters and print material placed around the hospital were suggested as a potential intervention strategy. Participants believed that this would be an effective way of encouraging safe lifting and handling techniques by serving as a useful reminder for nurses. Pre‐employment screening prior to nurses beginning work in any clinical context was also suggested as a strategy that could help to prevent injuries, by allowing healthcare organizations to identify nurses at greatest risk and to provide targeted support accordingly. Identifying and addressing hazards in the environment was considered a responsibility of employers and a technique that would help to develop a total safety culture in a workplace.

#### 3.5.2 Manual handling training

Manual handling training provided to nursing students in clinical contexts was proposed as a potential intervention strategy. Manual handling training in the clinical environment was also thought to be worthy of investigation among nurses who are already working. Participants stressed that manual handling training needs to be practical and believed that this would be more beneficial than simply watching a video. The effect of providing regular manual handling “refresher” courses on injury rates was also highlighted as worth examining. Again, it was recommended that refresher courses be practical to be effective.

Another manual handling intervention strategy that participants believed warranted further investigation was the implementation of manual handling champions on individual hospital wards. It was expressed that these champions would serve as good role models to the nurses, influencing their lifting practices and potentially reducing musculoskeletal injuries as a result. One participant also suggested investigating the effectiveness of a lift team comprised of orderlies, or other healthcare workers, who received specialist manual handling training and could be called on to perform lifts and transfers for nurses.

#### 3.5.3 Workflow

Workflow was considered important in the promotion of nurse safety, with poorly organized workspaces making it difficult to engage with injury prevention techniques. Therefore, changes in workflow that encourage easy access to equipment, and opportunities for teamwork and collaboration, may be worth investigating to determine whether they can reduce the prevalence of injury.

#### 3.5.4 Footwear

Providing nurses with adequate footwear was another recommended intervention strategy. A nursing participant noted that the appropriate type of footwear for each individual nurse could be determined by a podiatrist. Participants agreed that this would be an effective way to promote good posture and reduce pain and discomfort among nurses.

#### 3.5.5 Electric beds

Participants felt that providing electric beds would be one way to effectively prevent injuries among nurses. Manual beds were considered a risk factor for low back problems, requiring nurses to adopt awkward body postures and placing unnecessary stress on the back.

#### 3.5.6 Needs analysis

Participants noted that the performance of a needs analysis to determine where and for whom equipment and training is most needed could be a worthwhile intervention strategy. Particularly, they suggested investigating intervention effectiveness in wards or environments where the frequency of lifting and handling is high. Participants acknowledged that it is difficult for organizations to determine exactly how much equipment and support is needed for each individual ward.

#### 3.5.7 Stories

Finally, participants felt that the effect of sharing stories about experiences of injury could be evaluated. This was viewed as a potentially powerful method for keeping risk of injury at the forefront of nurses' minds. Sharing stories might also help nurses to gain a better understanding of the circumstances where injuries are most likely to occur.

## 4 DISCUSSION

While participants identified a number of strategies to prevent injuries among nurses, the use of these techniques was believed to be highly variable. In fact, there was some disagreement among participants with respect to how frequently manual handling and lifting equipment are used in clinical practice. However, participants consistently identified barriers to engagement with injury prevention strategies, which included the following: the availability of equipment, age, personal factors, contextual factors, staffing and time. Developing a culture of safety, manual handling training in clinical contexts, changes in workflow, ergonomic footwear, the provision of electric beds, needs analysis and the sharing of stories were additional approaches to preventing musculoskeletal injuries that participants considered worthy of investigation.

Although education was frequently identified as a strategy to prevent injuries, participants were concerned about the duration of education relating to musculoskeletal disorders was insufficient in NZ. There is evidence that limitations associated with education and training may contribute to nurse injuries. In a Canadian study of 1,645 nurses, 52.3% of the 416 who experienced back injuries attributed their injuries to inadequate training (Yassi et al., 1995). Furthermore, research suggests that education alone is not enough to prevent musculoskeletal injuries, not only in nurses (Harber et al., 1994) but also in other occupational groups (Daltroy et al., 1997).

Participants identified equipment and health and safety policy as additional strategies available to prevent injuries in NZ. The promotion of multiple approaches may be beneficial, given that sole use of mechanical equipment has not been found to reduce injury risk among nurses in the United States (Pompeii et al., 2009). However, of concern are international findings that even with an increased emphasis on education, equipment and “no lift” policies, nurses continue to sustain musculoskeletal injuries (Hunter et al., 2010). In a cross‐sectional survey of 897 undergraduate nursing students and 111 graduate nurses in Australia, participants reported high lifetime (79%), 12 month (71%) and 7 day (31%) rates of low back pain, which were largely attributed to bending or lifting, despite the implementation of multiple preventative strategies to reduce back injury (Mitchell, O'Sullivan, Burnett, Straker, & Rudd, 2008).

Having prevention strategies in place may not be sufficient to reduce the high rates of injury and pain, if implementing these strategies is problematic. A number of participants believed that the use of manual handling techniques and equipment was not often evident in daily practice. They also noted that despite “no lift” policies being in place, it is not always clear to nurses what the alternatives to lifting are. Research in the United States suggests that a large number of nurses are unaware of safe lifting guidelines (Vendittelli et al., 2016).

Numerous barriers to engagement with prevention techniques were identified. Perceptions of limited availability of equipment and inadequate staffing are consistent with findings of a survey of 435 registered nurses in Michigan (Vendittelli et al., 2016). Only 33% of participants considered staffing adequate for the performance of safe patient handling tasks and only 39% felt that enough lifting equipment was available. Both availabilities of equipment and staffing levels have been associated with the experience of injuries among nurses (Lee, Faucett, Gillen, & Krause, 2013; Trinkoff et al., 2003; Yassi et al., 1995). Participants in our study reported that ensuring adequate staff levels is one of the most effective ways to prevent injuries among nurses, a sentiment that has also been communicated by orthopaedic and intensive care nurses in Canada when asked how work‐related low back injuries could be reduced (Vieira, Kumar, Coury, & Narayan, 2006).

Age and other personal factors (including beliefs, selflessness and weight/physical fitness) were also identified by participants as barriers to nurses using injury prevention strategies. Overall, participants felt that older nurses are less inclined to use strategies to prevent injuries, largely because they are used to traditional methods of lifting and handling (such as a “do‐it‐yourself” approach). The altruistic intentions of many nurses have been recognized in the literature and acknowledged as a potential contributor to back injury (Gallagher, 2011); nurses may feel compelled to provide care to patients even when they are ill‐equipped or ill‐prepared. Being overweight or having low physical fitness was thought to reduce engagement with proper techniques to prevent injuries, which may explain why factors such as smoking, failure to exercise and body mass index have been associated with greater odds of experiencing a low back disorder among nurses and other workers (da Costa & Vieira, 2010).

Contextual factors, including demands from other staff members, hierarchies, workload and limited time, were also identified as barriers by participants. Hierarchies among staff in the hospital environment and work overload (related to inadequate staffing) were thought to contribute to heightened stress levels among nurses, reducing their capacity to implement prevention techniques. Stress has been found to have an important relationship with musculoskeletal disorders. In a survey of 53 U.S. nurses, self‐reported stress levels were higher in participants with a history of work‐related low back pain (73%) compared to those with no such history (Cato, Olson, & Studer, 1989). Other research involving 144 nurses from six Hong Kong district hospitals found that poor work relationships with colleagues were an independent risk factor for the onset of low back pain over a 12‐month period (Yip, 2004). The heavy workload and time pressures experienced by nurses have been well documented (Engels, Van Der Gulden, Senden, & van't Hof, 1996; Vieira et al., 2006), and systematic review findings have shown that workload and high psychosocial work demands are causally related to the development of work‐related musculoskeletal disorders (da Costa & Vieira, 2010), particularly among nurses (Menzel, Brooks, Bernard, & Nelson, 2004; Retsas & Pinikahana, 2000).

### 4.1 Relevance to clinical practice

Participants provided diverse suggestions regarding strategies to prevent musculoskeletal injuries among nurses that are worthy of further investigation or implementation in clinical practice. Fostering a culture of safety in work environments was a key theme under which several techniques were categorized, many of which address the barriers to safe practice that participants identified. Changes in management approaches (such as allowing nurses to be involved in decision‐making regarding health and safety processes) were recommended, which would likely have positive implications for the quality of workplace relationships. Regular assessments of competence were also endorsed. In NZ, ACC “Moving and Handling People Guidelines” recommend manual handling training and competence assessment for all new employees who are required to move and handle people, and when new equipment or work practices are introduced (Accident Compensation Corporation, ACC, 2011). However, the degree to which different healthcare organizations implement these guidelines is unclear and is an avenue for further research.

Participants recommended that manual handling training and assessments of competence take place in the clinical environment and not rely on online video training only. ACC guidelines state that employees must be given opportunities to practice the techniques they are taught and discourage passive learning (whereby staff simply watch videos), citing this as an ineffective way to provide manual handling training (ACC, 2011). However, our findings suggest that nurses are not always provided with such opportunities. Participants suggested that manual handling champions could be appointed on individual wards, with these nurses responsible for modelling safe patient handling practices, ensuring compliance with ergonomic equipment and creating an overall safety culture. While initiatives in the Netherlands (Knibbe, Knibbe, & Klaassen, 2007) and the United States (Stenger, Montgomery, & Briesemeister, 2007) report using this approach with some success, there are yet to be any studies published comparing the injury rates of nurses on wards with manual handling champions and those without.

The promotion of nurse well‐being on behalf of employers was an additional recommendation, contributing to the overall culture of safety in a workplace. Specifically, suggestions included encouragement to report injuries as early as possible and to engage in physical activity and relaxation. Although an earlier examination of 119 nurses did not find any differences in fitness and lifestyle characteristics between those who sustained injuries during the 18 month study period and those who did not (Ready, Boreskie, Law, & Russell, 1993), there is preliminary evidence that physical fitness can help reduce disability associated with low back pain in this occupational group (Warming et al., 2008).

Several additional strategies proposed to have potential to prevent musculoskeletal injuries and pain among nurses have yet to be investigated using rigorous study designs, including the effectiveness of print material strategically placed in healthcare settings to encourage safe practice and the use of pre‐employment screening. A Cochrane systematic review concluded that all research to date regarding pre‐employment examinations and implementation of measures to mitigate risks following the screening process is of very low quality (Mahmud et al., 2010). The use of stories to inform nurses of common circumstances and consequences of injury is also yet to be empirically investigated, despite storytelling having been cited as an effective educational tool (Davidhizar & Lonser, 2003). Implementation of a lift team comprised of individuals who have received extensive training and are adept at using mechanical lifting devices for performing patient transfers was another strategy suggested by participants, which has received relatively limited investigation. Nevertheless, an evaluation of 10 healthcare facilities reported decreases in number of back injuries and lost workdays among nursing personnel following implementation of a lift team intervention (Charney, 1997).

Issues with workflow in healthcare settings were highlighted by participants. This is consistent with previous findings, where 53% of a sample of 846 nurses in The Netherlands reported that the ergonomic layout of their workplace was disagreeable (Engels et al., 1996). Modifications to workplaces that encourage communication and teamwork and that allow nurses to easily source equipment would enhance workflow and have potential to reduce injury. Training in workstation adjustment has been significantly associated with a lower prevalence of back musculoskeletal disorders among nurses (Trinkoff et al., 2003). Participants also felt that ensuring nurses have appropriate footwear (as determined by a podiatrist) could help to minimize potential for injury. Evaluations of professional footwear have determined the types of footwear likely to provide the greatest degree of shin and ankle comfort for nurses (Chiu & Wang, 2007) and the potential for unstable shoes to reduce low back pain and disability among nurses (Vieira & Brunt, 2016) and other healthcare professionals (Armand et al., 2014) is beginning to be identified.

### 4.2 Limitations

A limitation of this study is that the perspectives of physiotherapy and nursing academics cannot be generalized to reflect the perspectives of nurses working in clinical contexts. It is possible that the academics in our sample do not have the same understanding of the unique challenges experienced by nurses and the strategies that would be most applicable in practice. Nevertheless, participants were selected because of their involvement in teaching or training nurses, so it is highly likely they are aware of the key contributors to injury in this group. Another limitation is that this research was conducted at one of NZ's 20 District Health Board regions. Perspectives on the issue may vary across regions of NZ and are unlikely to reflect those of individuals responsible for training nurses in other countries. A further limitation of the study is that it is not possible to comment on the effectiveness of the strategies recommended by participants. However, the purpose of this research was to investigate ideas regarding techniques that could help to prevent injuries among nurses, from the perspectives of individuals deemed to have expert knowledge in the area. Novel approaches to address the high rates of injury in this population are urgently needed in light of evidence that nurses currently accept musculoskeletal pain and injury as an inevitable consequence of their role (Boniface, Ghosh, & Robinson, 2016).

## 5 CONCLUSION

This appears to be the first study to explore physiotherapy and nursing academics' perspectives on strategies available to prevent injury among nurses, barriers to engagement with these strategies and potential approaches that are worthy of further investigation. While several strategies are available, numerous barriers make it difficult for nurses to implement them in clinical practice. Results indicate the importance of considering these barriers when designing interventions to increase their effectiveness.

## CONFLICT OF INTEREST

No conflict of interest has been declared by the authors.

## AUTHOR CONTRIBUTIONS

Amy Richardson and Gagan Gurung were responsible for data collection and the coding of each interview. Helen Harcombe and Sarah Derrett contributed to the development of a coding framework. Each author was involved in writing the manuscript and reviewing drafts.
